# Sensitivity and prognostic significance of circulating tumor DNA (ctDNA) in stage I to III malignant melanoma

**DOI:** 10.1007/s00432-026-06478-w

**Published:** 2026-05-09

**Authors:** Ann-Sophie Bohne, Marilena Heber, Franziska Axt, Nikolas von Bubnoff, Katharina Kähler

**Affiliations:** 1 Department of Dermatology, Venerology and Allergology, University Hospital Schleswig-Holstein, Campus Kiel, Kiel, Germany; 2 Department of Hematology and Oncology, University Hospital Schleswig-Holstein, Campus Lübeck, Lübeck, Germany

**Keywords:** Biomarkers, Melanoma, ctDNA, Survival, Recurrence

## Abstract

**Background:**

Risk stratification to guide adjuvant treatment in early stages of melanoma becomes increasingly important. This study investigated circulating tumor (ct)DNA in early melanoma stages to predict disease progression in real-world settings in German melanoma patients.

**Methods:**

In this retrospective, single-center study, 61 patients with melanoma stages I-III with known disease-progression following primary diagnosis were included. Patients were requested to have baseline serum samples ≤ 4 weeks after diagnosis and ≥ 12 weeks preceding disease progression. In ctDNA isolated from 185 serum samples matching inclusion criteria, BRAF V600E/K, NRAS Q61K/L/R and TERT promoter mutations were quantified and correlated with serum levels of S100 and LDH.

**Results:**

Mutated ctDNA was detected in ≥ 1sample in 43 of 53 patients (81.13%). CtDNA was more sensitive in the prediction of melanoma relapse than established biomarker S100, LDH (*p* < 0.001) and both LDH/S100 (*p* < 0.001). Highest mean ctDNA was measured during shift of stage III to stage IV disease (24.25 cps/µL) and shift within stage III (12.42 cps/µL). The detection of ctDNA at any time point trended towards shorter overall survival.

**Conclusion:**

Our study demonstrates the superiority of ctDNA harboring melanoma specific mutations over LDH and S100 in identifying patients at risk for recurrence in early melanoma stages in a single center cohort of melanoma patients. Future prospective trials are warranted to confirm this.

**Supplementary information:**

The online version contains supplementary material available at 10.1007/s00432-026-06478-w.

## Introduction

Malignant melanoma is one of the most common cancers in young adults (Buja et al. 2024; Keegan et al. 2024). Although the therapeutic landscape of melanoma changed dramatically during the past decade with new therapy modalities improving survival rates, patients with low-risk melanoma still experience disease progression (Schmid-Wendtner et al. 2003). To prevent melanoma recurrence, targeted therapies and immunotherapies are approved in the metastatic and recently adjuvant setting. Both treatment modalities are associated with specific toxicities which can occur temporarily but also can potentially lead to lifelong sequelae. Therefore, it is mandatory to improve the identification of patients at risk for melanoma recurrence and disease progression to assess the individual risk and thus grant access to effective therapies sooner for patients at risk and to avoid lifelong side effects in patients who are not at risk of disease progression or recurrence. Currently, the main criterion to stratify the risk of recurrence and to estimate survival is the classification of melanomas using tumor thickness of the primary tumor and the presence of metastasis to the lymph nodes, skin or distant organs (Long et al. 2023). In addition to skin examinations, imaging techniques e.g. ultrasound of lymph nodes, CT scans and serum markers lactate dehydrogenase (LDH) and S100 are used to detect melanoma recurrence or disease progression in advanced melanoma stages.

During the past years, liquid biopsy has been established. It involves the detection of circulating tumor cells (CTCs) and circulating tumor DNA (ctDNA) usually in blood samples of cancer patients. In melanoma patients, both CTCs and ctDNA have been detected and investigated regarding their potential as biomarker and predictor of disease progression and survival (Heidrich et al. 2023). Within ctDNA, tumor specific mutations, namely *BRAF*, *NRAS* and *TERT* Promoter mutations can be detected and quantified, and the amount of mutated ctDNA copies correlated with tumor volume and response to treatment (Braune et al. 2020). A prospective study of stage IIB, IIC and stage III melanoma showed a significant association of detection of pre-surgical ctDNA with later-stage and clinically evident stage III disease (Brunsgaard et al. 2023). In stage III and stage IV disease, we and others have shown that the detection of ctDNA was associated with shorter progression free survival (Braune et al. 2020; Marczynski et al. 2020). First emerging data in stage III melanoma patients identified detectable ctDNA as an independent predictor of progression free survival and distant metastasis free survival (Tan et al. 2019).

The significance of ctDNA in monitoring treatment response is currently being evaluated. First studies have shown, that ctDNA is a strong biomarker in patients with metastastic melanoma to indicate response as early as four weeks upon initiation of treatment, but sensitivity was decreased in monitoring intracranial disease activity (Braune et al. 2020; Khaddour et al. 2022; Lee et al. 2020). A case series suggested ctDNA as a potential tool to discriminate pseudoprogression from true progression in patients receiving immune checkpoint inhibitor-based therapy in case of inconclusive radiological findings as addition to imaging for response monitoring (Khaddour et al. 2022). Current trials evaluate the use of ctDNA to detect minimal resiudual disease and the impact of its detection on recurrence rates (Syeda et al. 2025).

To date limited data is available on the comparison of the established biomarkers S100 and LDH, and ctDNA with regard to tumor progression and overall survival in patients with early-stage melanoma (Ansstas et al. 2026). We therefore describe the real-world data of a single-center cohort of a German melanoma patients to add to the recently published data. The aim of this study was therefore to evaluate ctDNA in comparison to the established biomarkers in a retrospective cohort of stage I to III melanoma.

## Materials and methods

### Patients

Blood, tissue samples and pseudonymized patient data of melanoma patients have been collected and archived after obtaining written informed consent (Ethics vote D 431/06) in the dermato-oncological section of the department of dermatology of the university hospital Schleswig–Holstein, Campus Kiel. Serum samples were processed using a standardized protocol and stored long-term at − 80 °C.

Using the database, 61 patients were retrospectively identified diagnosed with localized malignant melanoma (AJCC Stages 2017: 4 patients in stage I, 20 patients in stage II, 37 patients in stage III) at initial diagnosis with disease progression during the following years. Only patients with known *BRAF* and *NRAS* mutational status of the primary tumor were included. Baseline serum obtained within of 8 weeks of primary melanoma diagnosis and follow-up samples obtained ≥ three months prior to diagnosis of disease progression had to be available. The samples had been obtained during follow-up visits at the physician’s discretion. Two cohorts of patients were formed, cohort 1 (32 patients) with *BRAF* or *NRAS* mutated malignant melanoma and cohort II (29 patients) with *BRAF* and *NRAS* negative primary tumor. *TERT* promoter mutation analysis had not been performed in any of the patients’ tissue and was exclusively performed on serum samples in cohort II. Additionally, a control cohort with serum from 11 stage IV melanoma patients was included to validate ctDNA detection. Patient selection and study group formation is displayed in Online Resource 1.

### Isolation and quantitation of ctDNA from serum

CtDNA was purified from 2 ml of serum using the QIAamp Circulating Nucleic Acid kit (QIAGEN, Hilden, Germany) according to the manufacturer’s instructions. DNA quantity was measured using the Bioanalyzer High Sensitivity DNA Reagents kit (Agilent Technologies Inc., Santa Clara, United States) following the manufacturer´s instructions. Assays for detection of *BRAF* and *NRAS* were used as described previously (Braune et al. 2020) and as displayed in Online Resources 4 and 5. Two individual droplet digital polymerase chain reaction assays for *TERT* promoter mutations C228T and C250T were designed and validated, but did not prove to be superior to the commercially available ones (Bio-Rad Laboratories GmbH, Munich, Germany), which were ultimately used. Droplets were generated and analyzed using the QX200 Droplet Digital PCR system (Bio-Rad Laboratories, Munich, Germany). Four wells per sample, each containing 7 μL of eluted cell-free ctDNA, were analyzed. QuantaSoft Version 1.7.4.0917 (Bio-Rad Laboratories) was used for data acquisition and analysis. The limit of blank, limit of detection, and lowest detectable copy ratio were determined for each assay as reported previously for BRAF and NRAS (Braune et al. 2020), and in Online Resource 6 for *TERT* promoter mutations.

### Statistical analysis

Descriptive statistics was used to calculate mean, median, standard deviation and frequencies of the obtained parameters. 5% trimmed mean values were calculated to exclude the 5% lowest and highest outliers and compared to calculated mean values. Measurements of S100 > 0.10 µg/l and LDH > 250 U/l were regarded as positive and detection of *BRAF*, *NRAS* or *TERT* promoter mutated ctDNA was regarded as ctDNA^+^ probe. Regarding the comparison of the three biomarkers ctDNA, LDH and S100 only sensitivity was tested, testing specificity was not possible. Statistical significance regarding the sensitivity of the three biomarkers was tested using x^2^-test including Yates correction. Progression-free survival (PFS), defined as date of primary melanoma diagnosis to first diagnosis of disease progression or death and overall survival (OS) defined as date of primary melanoma diagnosis to date of death or last follow-up was calculated. Survival curves were generated using the Kaplan–Meier method. When comparing OS and PFS of ctDNA^−^ and ctDNA^+^ samples, Hazard ratios (HRs) and 95% confidence intervals (CIs) of the HRs for PFS or OS were derived from Cox proportional hazards models. Statistical analysis as was performed using R (R Development Core Team 2021) (version 4.5.2, which was also used to generate the figures included.

## Results

A total of 61 patients with 185 plasma samples and matched levels of S100 and LDH.

were included. The mean number of samples per patient was 3 (range 2–5). Of 61 patients, 32 patients (52.5%) had tissue BRAF or NRAS mutations. Of these, 14 of 21 patients (66.7%) with tissue BRAF V600E were ctDNA^+^ for BRAF V600E at least at one time point. Three of four patients (75%) with tissue BRAF V600K and five of seven (71.4%) patients with tissue NRAS mutation were ctDNA^+^ at least once. Of 29 tissue BRAF^−^ and NRAS^−^ patients, 21 patients (72.4%) were plasma TERT ctDNA^+^ at least once. Eight patients with 22 serum samples were tissue negative for BRAF and NRAS, had negative TERT ctDNA and were excluded from further analysis. Excluded patients did not have elevated LDH and only one sample (4.5%) had a matched positive S100 sample. AJCC stages of these ctDNA^−^ patients at the initial time of diagnosis were 1 stage IB, 3 stage IIB, 2 stage IIIB and 2 stage IIIC. Characteristics did not significantly differ from the remaining cohort. Two of these eight patients had exlusively distant metastasis of the central nervous system (CNS) and two patients also developed metastasis to CNS during disease progression. Of the remaining 53 patients, 163 blood samples were analyzed. The patient characteristics of these 53 are summarized in Table 1. CtDNA was detected in 65 samples (39.9%) from 43 patients (81%), with a mean number of positive samples per patient of 1.02 (range 0 to 4). Regarding AJCC 2017 stages, patients were distributed as follows: 3 patients in AJCC stage I, 17 patients in AJCC stage II and 33 patients in AJCC stage III.

**Table 1:** Study cohort and distribution of sex, age, AJCC stage at initial diagnosis and type of mutation. Study cohort (n = 53)

Characteristics	
*Age (years)*	
Mean (Range)	60.4 (23.0–84.0)
*Sex, n (%)*	
Female	24 (45%)
Male	29 (55%)
*AJCC stage at primary diagnosis, n (%)*	
IA / IB	3 (5.7%)
IIA / IIB	11 (21%)
IIC	6 (11%)
IIIA	7 (13%)
IIIB	7 (13%)
IIIC	15 (28%)
IIID	4 (7.5%)
*Type of tissue mutation, n (%)*	
Wildtype	21 (40%)
BRAFV600E	21 (40%)
BRAFV600K	4 (7.5%)
NRAS Q61K	3 (5.7%)
NRAS Q61L	3 (5.7%)
NRAS Q61R	1 (1.9%)
*Tumour thickness of primary tumour, (mm)*	
Mean	4.6 (SD 4.6)
Range	0.6–24.0

### Biomarker level depending on AJCC stage

Of the 163 samples screened for ctDNA, S100 levels were available in 151 samples and LDH in 152 samples respectively. CtDNA and levels of S100 and LDH were matched with the corresponding AJCC stage at the time of measurement. CtDNA was detectable in all AJCC stages. CtDNA^+^ patients were distributed as follows at initial melanoma diagnosis: 3 of 3 (100%) patients in AJCC stage I, 12 of 17 (70.6%) patients in stage II and 28 of 33 (84.9%) patients in AJCC stage III. Mean ctDNA levels were highest in stage III disease (18.74 cps/µL), followed by stage II (4.32 cps/µL), stage IV (2.62 cps/µL) and stage I disease (2.52 cps/µL). The same trends were visible when 5% trimmed mean values were regarded: stage III 5.04 cps/µL, stage II 3.18 cps/µL, stage IV 2.62 cps/µL and stage I 2.52 cps/µL. Patients with elevated S100 (≥ 0.11 µg/l) were distributed as follows at initial melanoma diagnosis: 2 of 3 (66.7%) patients in AJCC stage I, 8 of 17 (47.1%) patients in stage II and 10 of 33 (30.3%) patients in AJCC stage III. Mean S100 levels were highest in AJCC stage III (0.23 µg/l), 5% trimmed mean was highest in stage IV disease (0.12 µg/l). In contrast, neither mean LDH levels nor 5% trimmed mean levels were elevated in any of the samples from all AJCC stages (Table 2).

**Table 2:** Distribution of AJCC stages, corresponding S100, LDH and ctDNA measurements and distribution of biomarkers amongst patients

	S100B		LDH		ctDNA	
AJCC stage	Number of samples (positive samples, proportion of positive samples %)	Mean level, µg/l (Range); 5% trimmed mean	Number of samples (positive samples, proportion of positive samples %)	Mean level, U/l (Range); 5% trimmed mean	Number of samples (positive samples, proportion of positive samples %)	Mean level, cps/µL (Range); 5% trimmed mean
Stage I	5 (2, 40%)	0.11 (0.05–0.25); 0.11	5 (1, 20%)	205.00 (174–270); 205.00	6 (2, 33%)	2.52 (0.00–11.62); 2.52
Stage II	39 (8, 21%)	0.09 (0.03–0.44); 0.08	38 (3, 7.9%)	198.84 (140–305); 197.53	39 (14, 36%)	4.32 (0.00–50.54); 3.18
Stage III	95 (19, 20%)	0.23 (0,02–8,06); 0,09	96 (10, 10%)	204.65 (118–731); 191.88	105 (46, 44%)	18.74 (0,00–688,43); 5.04
Stage IV	12 (2, 17%)	0.12 (0.03–0.70); 0.12	13 (1, 7.7%)	186.69 (145–259); 186.69	13 (3, 23%)	2.62 (0.00–27.98); 2.62
Total number of samples	n = 151		n = 152		n = 163	
Detection of biomarker among patients n = 53						
Negative (%)	30 (57%)		41 (77%)		10 (19%)	
Positive (%)	23 (43%)		12 (23%)		43 (81%)	

The distribution of biomarkers with matched AJCC stages are summarized in Table 2.

#### Subgroup analysis regarding the distribution of ctDNA positive samples depending on AJCC stage

**Fig. 1:**
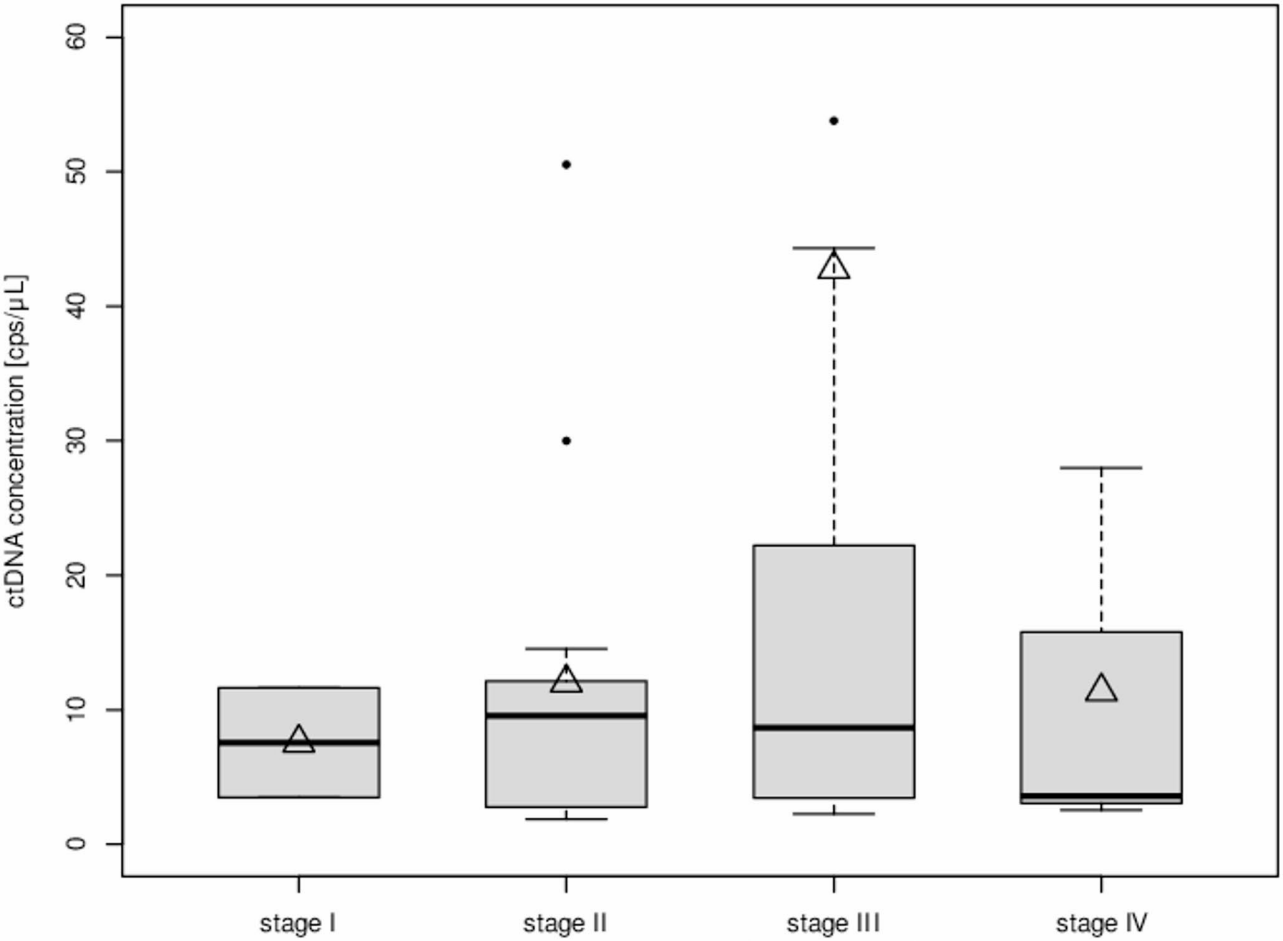
Concentration of ctDNA in positive samples and distribution regarding AJCC stages

Subgroup analysis of the 65 ctDNA^+^ samples was performed regarding distribution of AJCC stages. Two of five samples (40%) obtained in stage I disease were positive with a mean ctDNA concentration of 7.6 cps/µL (range: 3.5–11.6 cps/µL), 14 of 39 samples (36%) in stage II disease (mean ctDNA 12.0 cps/µL (range: 1.9–50.5 cps/µL), 46 of 95 samples (48%) in stage III disease (mean ctDNA 42.8 cps/µL (range 2.3–688.4 cps/µL)) and three of 12 samples (25%) in stage IV disease (mean ctDNA 11.4 cps/µL (range 2.5–28 cps/µL)). The results are displayed in corresponding box plots in Fig. 1. Additional data is displayed in Online Resource 2 regarding the biomarker distribution for biomarker positive patients and overlap in biomarker positivity.

Concentration of ctDNA in ctDNA positive samples dependent on the stage of disease at the time of obtaining the serum sample. The median is indicated in bold; the mean is indicated using an arrow head. Highest measured ctDNA levels were observed in stage III disease.

### Comparison of sensitivity of ctDNA, S100 and LDH

**Fig. 2:**
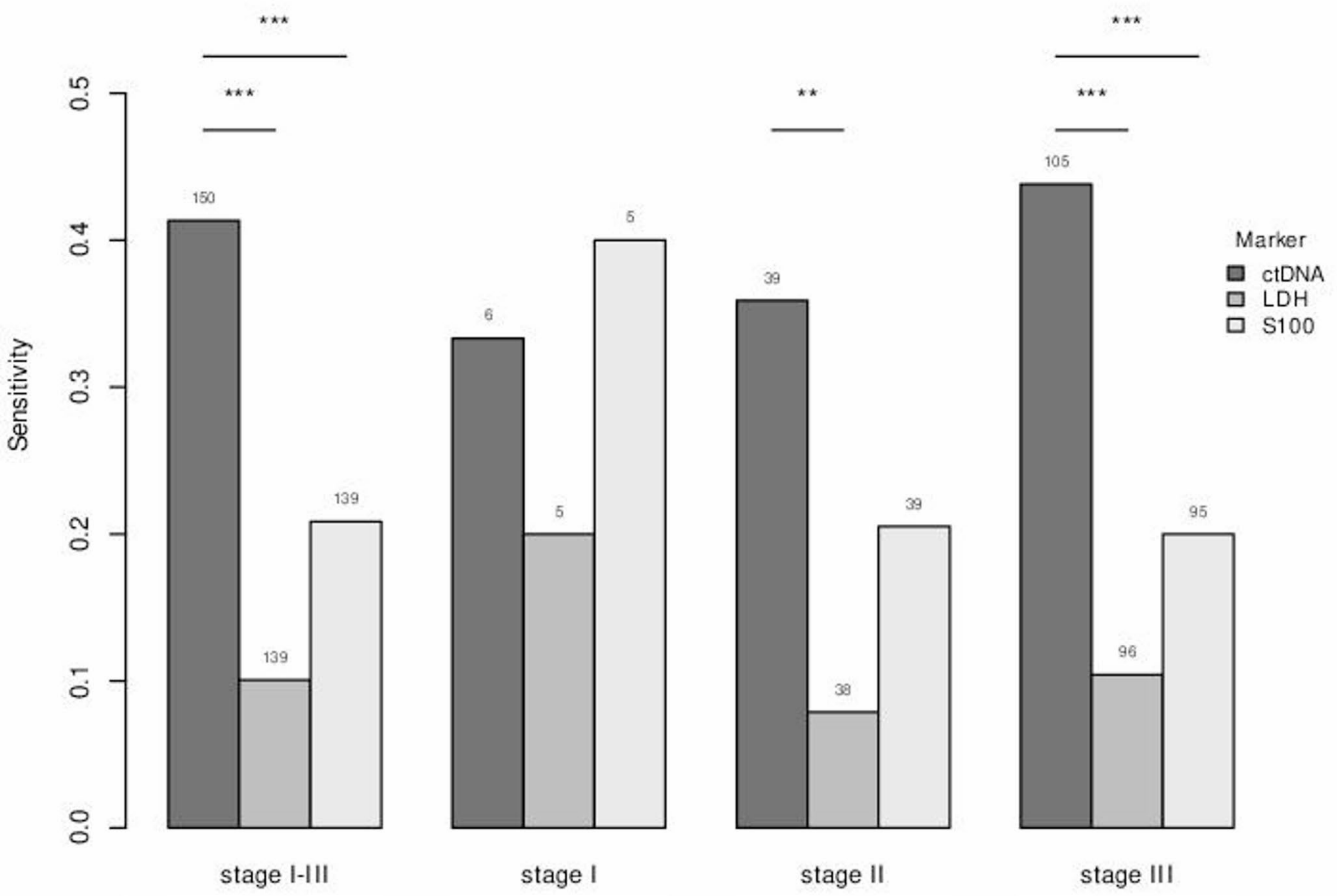
Comparison of sensitivity of S100, LDH and ctDNA in early stages of melanoma

The sensitivity of ctDNA was compared with the established biomarkers S100 and LDH in stages of limited disease (AJCC stages I and II respectively) and locoregional disease (AJCC stage III) and tested regarding statistical significance. In AJCC stage I no statistically significant difference regarding the sensitivity of the three biomarkers was observed due to low number of cases (n = 5). In stage II disease, sensitivity of ctDNA was superior to LDH (*p* < 0.01) with no statistical significance when comparing ctDNA and S100. In locoregional limited disease (AJCC stage III) ctDNA was significantly more sensitive than S100 and LDH (*p* < 0.001). The sensitivity of ctDNA was significantly superior to S100 and LDH, even when all three early disease stages (stage I, II and III) were considered collectively (*p* < 0.001). The results are shown in Fig. 2.

Comparison of the sensitivity of biomarkers S100 (light grey), LDH (medium grey) and ctDNA (dark grey) during all early stages of disease (I-III) and separately for each stage. Numbers above each collumn indicate the number of samples. Only elevation of LDH > 250 U/l and S100 > 0.1 µg/l were taken into account. X^2^-test was performed to compare ctDNA with LDH and ctDNA with S100 and statistical significance was registered (∗ : *p* < 0.05, ∗  ∗ : *p* < 0.01, ∗  ∗  ∗ : *p* < 0.001).

### Levels of ctDNA and AJCC stage shift

Serum samples were collected from patients with known progression from localized disease stages (AJCC I, II) to AJCC stages associated with locoregional disease (AJCC stage III) and/or to a distant metastastic disease stage (AJCC stage IV). All samples were obtained at least 12 weeks prior to further examinations (MRI, CT scan, ultrasound and full body examination) revealing disease progression. Concentrations of ctDNA varied depending on the type of progression from localized disease (AJCC stages I, II), to locoregional metastasis (AJCC stage III) and to distant metastasis (AJCC stage IV). 27 samples were obtained prior to progression from localized disease to locoregional metastasis, 18 samples were obtained prior to progression from localized disease to distant metastasis, 54 samples were obtained prior to progression from locoregional metastasis to distant metastatic disease, 64 samples were obtained prior to stage shift within stage III or within stage IV disease. Different types of stage shifts, associated sample sizes and ctDNA concentrations are displayed in Table 3.

**Table 3:** ctDNA concentrations in all samples depending on stage shift during disease progression

AJCC stage Shift	n	Mean (cps/µL)	5% trimmed mean	Range
Stage I to III	4	0.87	0.87	0.00–3.49
Stage II to III	23	2.79	2.37	0.00–14.52
Stage I to IV	2	5.81	5.81	0.00–11.62
Stage II to IV	16	6.51	6.51	0.00–50.54
Within stage III	53	12.42	3.37	0.00–354.36
Stage III to IV	54	24.25	8.66	0.00–688.43
Within stage IV	11	3.10	3.10	0.00–11.62

When regarding only ctDNA^+^ samples 6 months prior to the diagnosis of progression of the disease, this trend is still visible as displayed in the figure shown in Online Resource 3.

### ctDNA lead time regarding time to progression

**Fig. 3:**
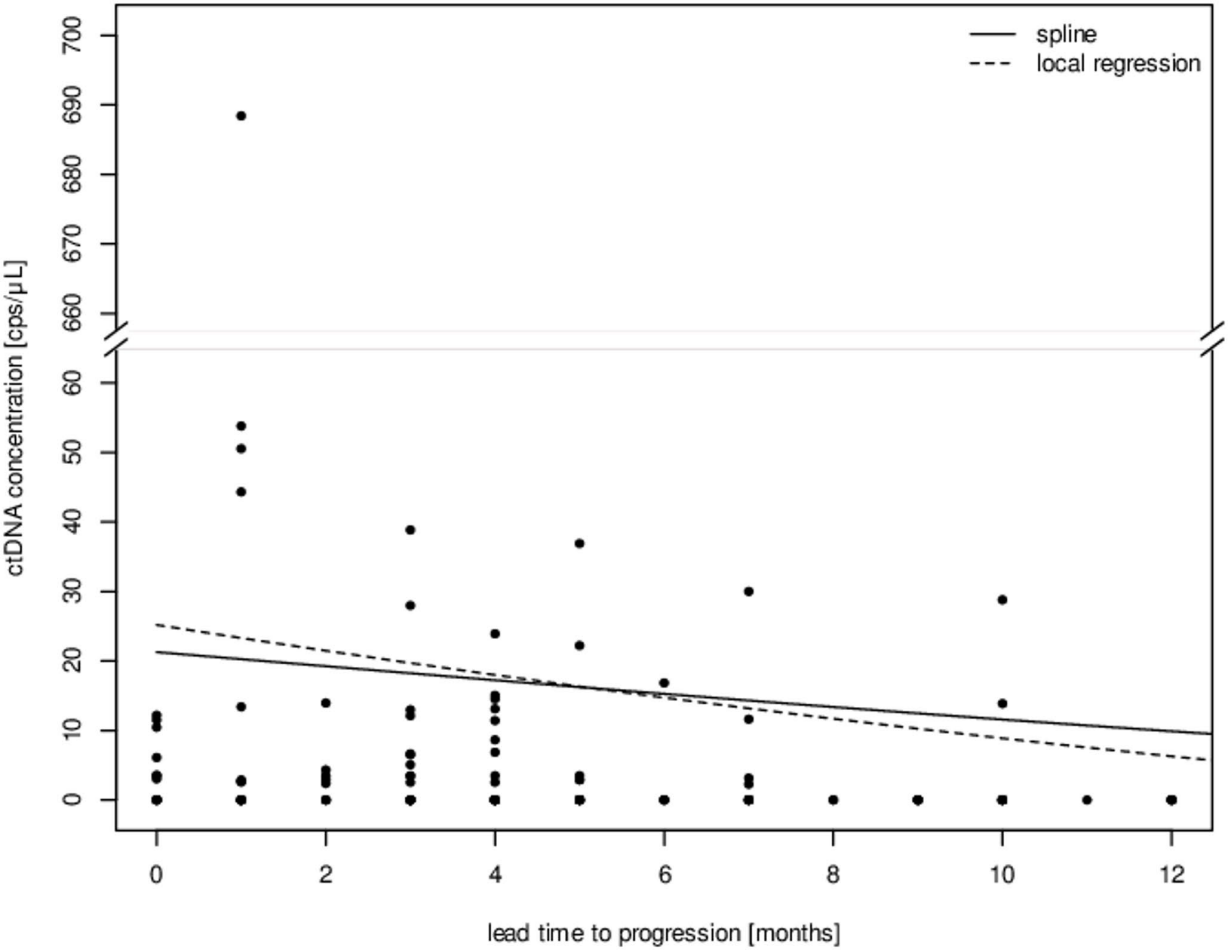
Average ctDNA concentration in the months prior to diagnosis of disease progression

We next analyzed the relationship between ctDNA detection and diagnosis of disease progression through regular follow-up examinations of the 163 samples and subsequent stage shift. We observed higher ctDNA concentrations in a period of 6 months prior to clinical disease progression, as displayed in Fig. 3.

Average ctDNA concentration 12 months prior to disease progression including local regression (dotted black) and spline smoother (black). We used an interrupted y-axis to include ctDNA outliers.

### Sensitivity of ctDNA and established biomarkers S100 and LDH to detect subsequent disease progression

**Fig. 4:**
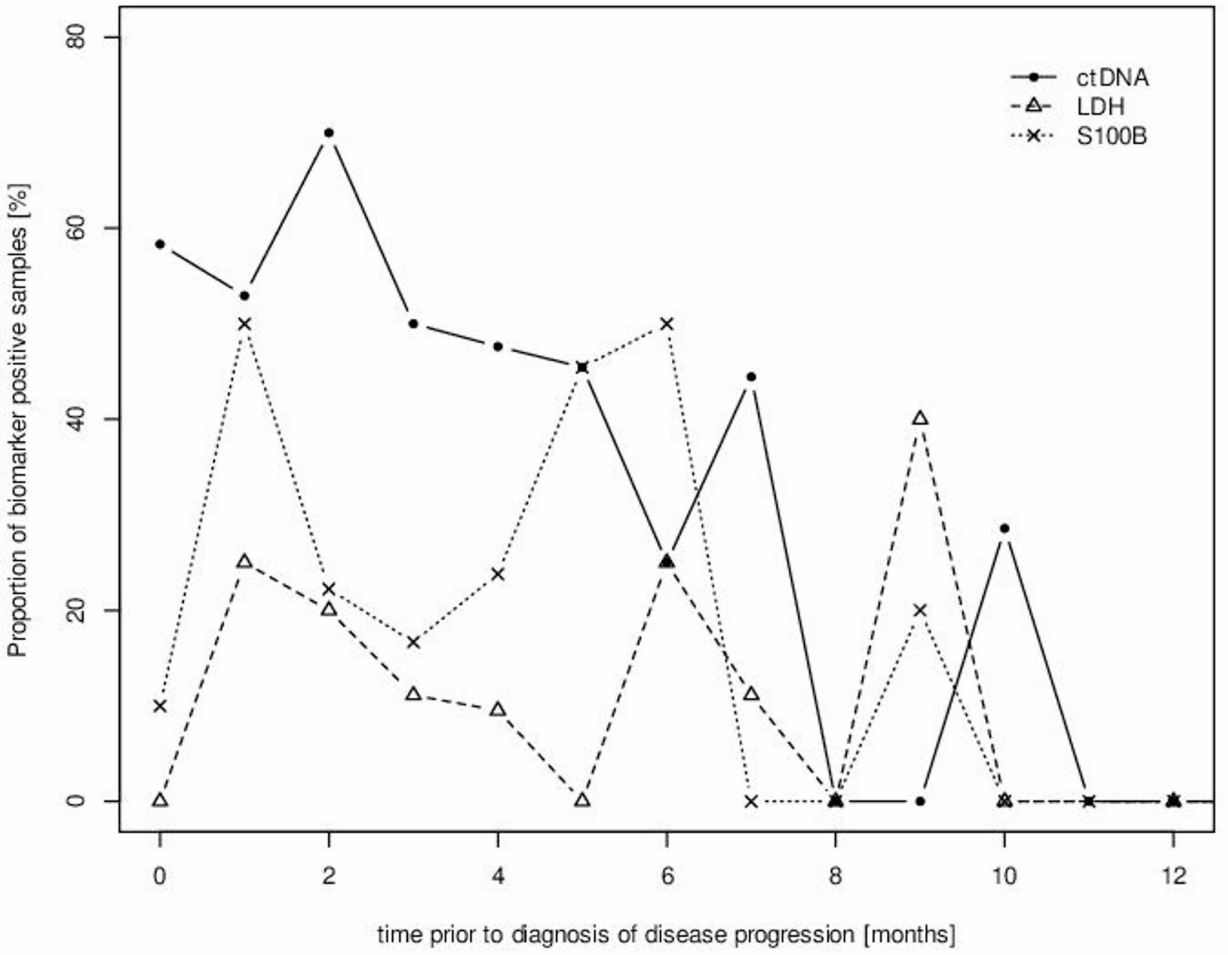
Positivity of biomarkers and time prior to diagnosis of disease progression

Positive samples of ctDNA, S100 and LDH were matched to subsequent disease progression by correlating marker positivity and the timeframe prior to clinical disease progression. A higher proportion of samples were ctDNA^+^ compared to samples with elevated S100 and LDH in a period of six months prior to disease progression, and the proportion of elevated LDH and S100 samples remained stable throughout the 12 months preceding disease progession. The results are displayed in Fig. 4.

Proportion of positive samples of each biomarker dependent on the time preceeding diagnosis of progression (months). Sample positivity was defined as measurable ctDNA, elevation of LDH ≥ 250 U/l and S100 ≥ 0.11 µg/l.

### Correlation of biomarkers and patient characteristics

**Fig. 5:**
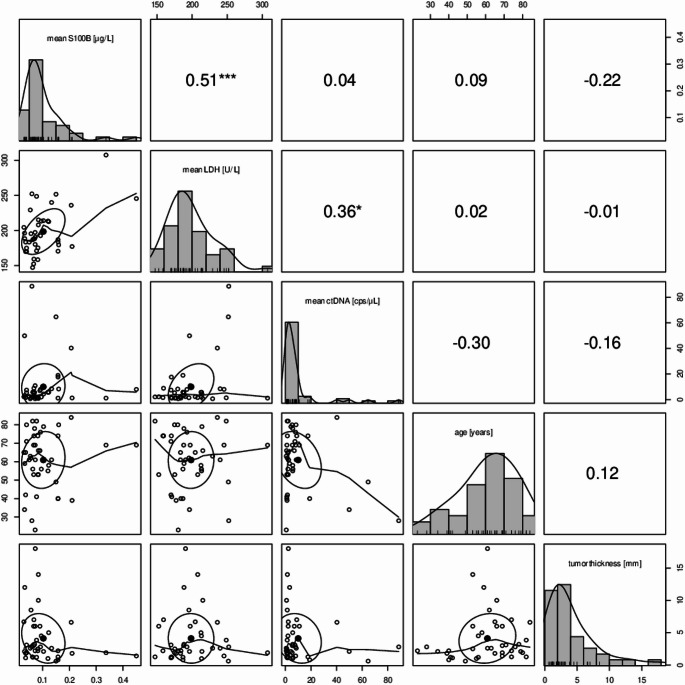
ctDNA positive patients and correlation with clinical characteristics

In ctDNA^+^ patients, ctDNA, S100 and LDH were correlated with age and tumor thickness. A correlation was found for LDH and S100 (r = 0.51; *p* < 0.001) and levels of ctDNA and LDH (r = 0.36, *p* < 0.05), whereas no linear correlation was observed between ctDNA and S100 (r = 0.04). A trend for a negative linear correlation could be observed for age and ctDNA (r = − 0.30) and thickness of the primary tumor and S100 (r = − 0.22), respectively. Results of the correlation analysis are displayed in Fig. 5.

Scatterplot matrices with consecutive histograms and correlations regarding all patients with detectable ctDNA at any point in time. Horizontal rows represent either mean S100, mean LDH, mean ctDNA, age or thickness of primary tumor. Each intersection of a horizontal row and a vertical row in the right upper half depicts the correlation coeffizient r and statistical significance indicated bei aterisks (∗ : *p* < 0.05, ∗  ∗ : *p* < 0.01, ∗  ∗  ∗ : *p* < 0.001). Each intersection of a horizontal row and a vertical row in the left lower half depicts the correlation of the two variables graphically.

### Detection of ctDNA and survival

Median OS of all 53 patients was 61 months (95% CI, 51 to 83 months) with the estimated median OS declining over time from 96% after 12 months (95% CI, 91 to 100%), to 81% (95% CI 71 to 92%) after 36 months and 27% (95% CI 16 to 45%) after 120 months. Median progression-free survival was 4 months (95% CI, 3.0 to 5.0 months) the progression-free survial rate after 3 months was 60% (95% CI, 49% to 75%), after 6 months 28% (95% CI, 18% to 43%), after 12 months 5.7% (95% CI, 1.9% to 17%), after 18 months 1.9% (95% CI, 0.3% to 13%) and 0% after 24 months.

**Fig. 6:**
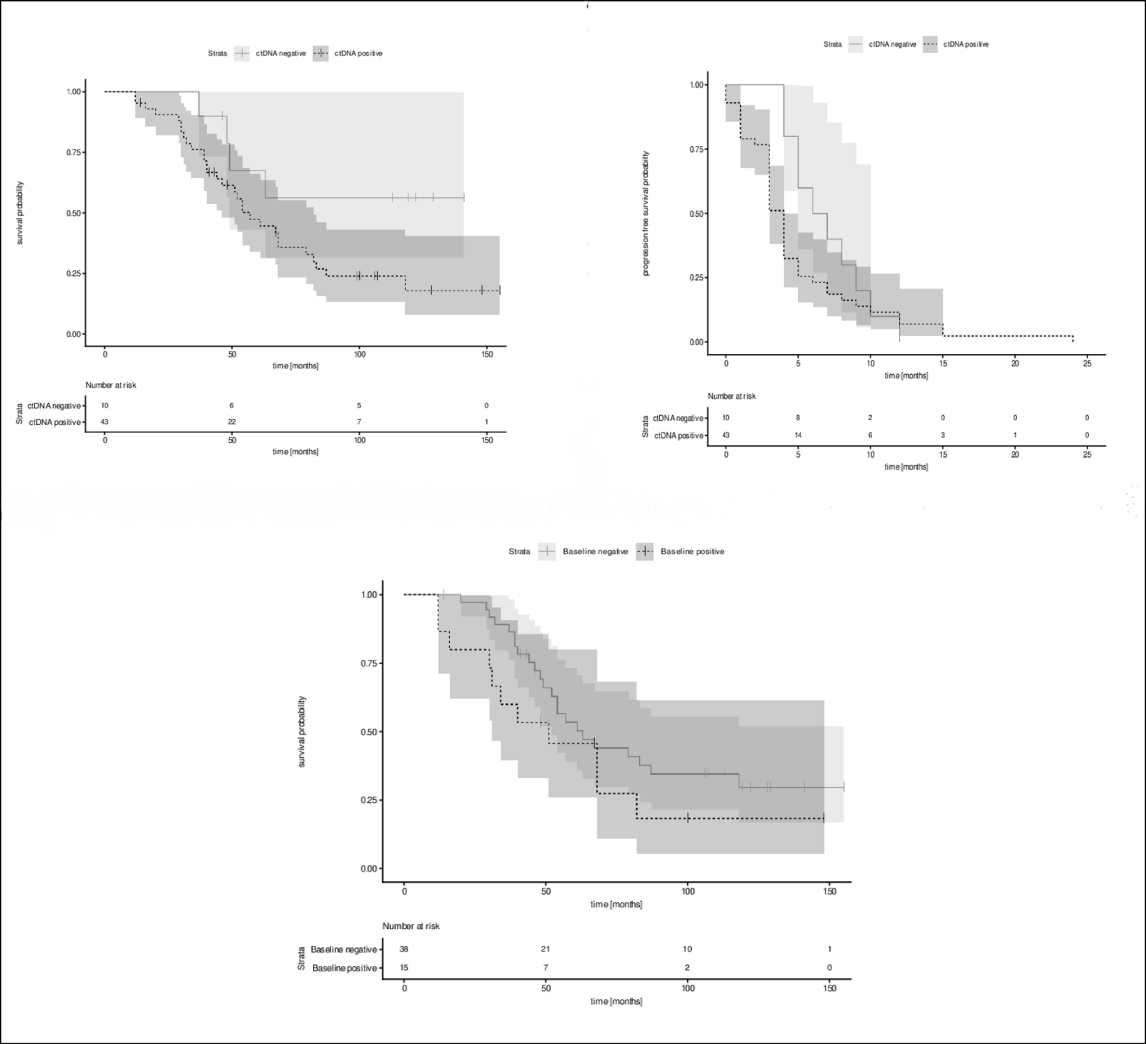
Overall survival

OS in patients with ctDNA^+^ samples at any time was 57 months (95% CI 46.82 months). Figure 6A depicts the Kaplan–Meier curves regarding OS in ctDNA^+^ and ctDNA^−^ patients. Cox-Regression analysis showed a hazard ratio of 2.56 (95% CI, 0.90 to 7.31) although these results did not reach statistical significance (*p* = 0.079).

Similarly, patients with detectable ctDNA showed a shorter progression-free survival of 4.0 months (95% CI 3.0 to 5.0 months) than patients with no detectable ctDNA at any point in time (PFS 6.5 months) with a Hazard ratio of 1.55 (95% CI, 0.77 to 3.12), but the results did not reach statistical significance (*p* = 0.22) (Fig. 6B).

The impact of ctDNA positivity on survival was weaker if subgroup analysis of patients with detectable ctDNA at baseline was performed (HR 1.59; 95% CI, 0.77 to 3.28; *p* = 0.2). Kaplan–Meier curves of this subgroup are shown in Fig. 6C. Correspondingly the impact of ctDNA positivity was weaker if baseline ctDNA positivity was regarded (HR 1.27; 95% CI 0.69 to 2.31; *p* = 0.44). Similarly, a tendency for a worse OS could be observed when ctDNA patients were grouped into ctDNA > 10 cps/µL and ≤ 10 cps/µL at with detectable ctDNA at any point in time (HR 2.36; 95% CI, 0.71 to 7.81; *p* = 0.16). This observation showed a weaker tendency in patients with detectable ctDNA at baseline (HR 1.23; 95% CI, 0.58 to 2.57; *p* = 0.59).

Figure 6A depicts the Kaplan–Meier curves regarding overall survival in ctDNA-positive and ctDNA-negative patients. Figure 6B depicts the Kaplan–Meier curves regarding progression-free survival in ctDNA positive and ctDNA negative patients. Figure 6C depicts the Kaplan–Meier curves regarding overall survival in the subgroup of patients with detectable ctDNA at baseline.

## Discussion

Despite improved adjuvant treatment options, reliable markers for early detection of recurrence of disease are still lacking. Although S100 and LDH are established in follow-up care, both biomarkers harbour a diagnostic gap in identifying patients at risk for progression. We therefore investigated ctDNA as a possible additive marker addressing this issue and compared it with S100 and LDH in early stage, non metastatic melanoma. We have previously shown that ctDNA has superior sensitivity compared to LDH and S100 in a cohort of stage III and stage IV patients (Braune et al. 2020). In this retrospective cohort of early stage melanoma, our data demonstrate superior sensitivity of ctDNA to detect active disease when compared to S100 and LDH. Direct comparison of the sensitivity of the three biomarkers ctDNA, S100 and LDH showed a higher sensitivity for ctDNA than LDH already in stage II disease, and a significantly higher sensitivity for ctDNA compared to S100 and LDH in stage III disease. Similar to our findings in stage II disease, Boerlin et al. demonstrated that elevated S100 levels correlated better with detectable ctDNA than LDH (Boerlin et al. 2022).

A comparison of the mean values for S100, LDH and ctDNA, and the corresponding AJCC stages shows that mean and 5% trimmed mean LDH levels were not elevated in any stage of melanoma in our retrospective cohort, hinting at the known lack of sensitivity of LDH in detecting disease progression in early melanoma stages. Interestingly mean S100 levels were also elevated in stage I serum samples and were only marginally elevated in stage IV samples. Although this finding could be due to the small sample size, it could also indicate that the dynamics of S100 elevation and only to a limited extent the actually measured value of S100 indicate progression or recurrence of the disease in early stages of melanoma. Similar observations have been made by Ertekin et al. showing, that during follow-up of high-risk melanoma patients, rising serum S100B values within the normal range can indicate disease progression (Ertekin et al. 2020). Interestingly mean ctDNA levels increased in a linear fashion from stage I to stage III and dropped in stage IV disease. Of note, also mean LDH and S100 values dropped from stage III to stage IV samples, suggesting a systematic effect. This could be due to the small sample size of isolated cerebral metastases in our cohort (2 of 10 stage IV patients) and the lack of sensitivity of biomarkers for intracranial disease (Lee et al. 2020). Trimmed mean values for S100 did not show the drop described, this in turn underlines the importance of ensuring that extreme outliers do not obscure generated data. In our cohort progressive disease from stage III to stage IV with exclusive progression of intracranial disease was not sufficiently detected by ctDNA (9 samples, 1 ctDNA positive sample), a similiar observation was made in patients with stage IV disease and only intracranial progressive disease (4 samples, no ctDNA^+^ sample). Although the sample size is small, this observation underlines a potential lack of sensitivity in detection of intracranial disease.

In order to assess AJCC stages and progression patterns where ctDNA adds information to established biomarkers and clinical follow-up, we analysed the average ctDNA concentration at the time of disease progression and the corresponding AJCC stage. Mean ctDNA concentration at the time of progression was highest in patients progressing within stage III (12.42 cps/µL) or in patients progressing from stage III to stage IV (24.25 cps/µL), and was lower upon progression from localized stages I or II to locoregional metastasis stage III (0.87 and 2.79 cps/µL, respectively). Interestingly, patients without stage shift in stage IV displayed lower mean ctDNA level (3.1 cps/µL), suggesting that increased ctDNA values indicate aggressive tumor biology and risk for progression and stage shift. Further differentiation between lymph node metastasis or subcutaneous and cutaneous metastasis without nodal involvement was not examined in the study population, and subcutaneous disease sites as well as intracranial disease sites are potentially not sufficiently monitored using ctDNA (Wong et al. 2017). These obersvations differed when extreme shedders were excluded using the trimmed mean, the highest trimmed mean was observed in progression from stage III to stage IV (8.66 cps/µL), followed by progression from stage II to stage IV (6.51 cps/µL) and stage I to stage IV (5.81 cps/µL). Even after exclusion of outliers, progression within stage III was still associated with higher ctDNA trimmed mean values than progression within stage IV disease (3.37 cps/µL vs. 3.10 cps/µL). This could be due to the aforementioned reasons or the study design, which was primarily focused on ctDNA values preceding disease progression rather than ctDNA values during course of disease in late-stage melanoma patients.

To evaluate the benefit of ctDNA in the early detection of tumor progression, we measured the lead time between detection of an increase in ctDNA and clinical progression. The concentration of ctDNA increased up to six months prior to clinical disease progression. Similar observations were made by Brunsgaard et al. who found detectable ctDNA levels presurgically especially in patients in stage III disease (Brunsgaard et al. 2023). In this aforementioned study 20 patients were followed up by ctDNA and 30% (6 patients) of these 20 patients experienced disease recurrence, detection of ctDNA preceded the clinical recurrence in 50% of these 6 patients (3 patients) (Brunsgaard et al. 2023). When comparing ctDNA with LDH and S100 prior to clinical disease progression the proportion of ctDNA^+^ samples was higher, than the proportion of samples displaying elevated S100 or LDH, and high ctDNA levels occured during six months preceding disease progression. The superior sensitivity of ctDNA over LDH in the detection of non-RECIST disease progression is in line with previous observations (Chang et al. 2016; Le Guin et al. 2021; Stadler et al. 2022). Polivka et al. (2024) reported that the best prediction of melanoma recurrence was achieved when the presurgical and postsurgical detection of high BRAF^+^ ctDNA and elevated S100 was combined.

When investigating the dependence of the biomarkers with other patient characteristics, we did not find a significant linear correlation between S100, LDH and ctDNA. The lack of correlation of these markers underlines the potential benefit of ctDNA as an independent tumor marker in patient monitoring indicating aggressive biology. We observed a negative correlation between detection of ctDNA and patient age, which could be due the presence of a less agressive tumor biology with low ctDNA shedding in older patients.

Detection of ctDNA seems to impact OS and similar results have been reported by Lee et al. in a group of patients with high-risk stage II and stage III melanoma (Lee et al. 2018).

The correlation of ctDNA detection and OS showed a trend in a negative effect of ctDNA positivity on survival, although these findings did not reach statistical significance. A meta-analysis by Gandini et al. (2021) underlines these findings, since patients with detectable ctDNA before treatment had worse PFS and OS, and ctDNA positivity during follow-up was also associated with adverse PFS and OS. The potential of monitoring patients receiving adjuvant therapy has been evaluated by Marchisio et al. who were able to show that baseline positivity for ctDNA was a negative predictor for survival and longitudinal ctDNA detection during adjuvant treatment reflected the clinical outcomes of patients (Marchisio et al. 2024). Similar observations were currently published by Syeda et al. who examined BRAF mutant ctDNA in stage III melanoma patients receiving adjuvant therapy. In this study patients with baseline ctDNA positivity as well as molecular relapse during adjuvant treatment showed shorter median recurrence free survival (Syeda et al. 2025). Although our findings regarding baseline positivity and overall survival differ, they do not contradict the previously described results since our examined samples were obtained in a period of time when adjuvant treatment options in melanoma were limited to interferon. Chan et al. were able to demonstrate that the detection of postsurgical ctDNA and lack of ctDNA clearance was associated with disease recurrence in stage III melanoma patients (Chan et al. 2024). This is in line with our findings of a tendency towards a shorter progression-free and overall survival in patients with detectable ctDNA at any point in time, although our results did not reach statistical significance.

Another potential use of ctDNA which should be investigated in the future, is its use in early response monitoring, because clearance of ctDNA after baseline detection and initiation of treatment is associated with response and favourable prognosis (Bratman et al. 2020; Braune et al. 2020). In stage IV disease the detection of ctDNA prior to comencing treatment is correlated with worse overall survival, a trend which we observed as well, although it did not reach statistical significance, probably due to the small sample size (Gandini et al. 2021).

In summary, our results show that ctDNA is detectable in early stages of malignant melanoma, displays superior sensitivity over LDH and S100, and detectable ctDNA can preceed clinical disease progression by up to six months. Thus, ctDNA could serve as a valuable addition to established biomarkers. Future challenges for integration into everyday clinical practice seem to be the dependence of the concentration of ctDNA on the localization of metastasis, standardization of detection and the lack of prospective data investigating clinical decision making stratified by ctDNA positivity in early-stage melanoma.

## Supplementary information

Below is the link to the electronic supplementary material.

Supplementary Material 1 Patients were divided into two subgroups. One subgroup including patients with BRAF or NRAS mutated primary tumors and one subgroup with wildtype BRAF and NRAS primary tumors. Following purification of ctDNA the BRAF+/NRAS+ subgroup samples were examined for presence of BRAF+/NRAS+ctDNA. Following purification of ctDNA the wildtype subgroup samples were examined for presence of TERT promoter mutated ctDNA.

Supplementary Material 2 Each biomarker (ctDNA, S100, LDH) is indicated by a circle. Overlapping areas of the circles of the diagram represent overlap in biomarker positivity in the indicated number of patients.

Supplementary Material 3 Strip chart depicting the logarithmic (LOG) concentration of ctDNA (y-axis) dependent on shifts between AJCC stages (x-axis). Only samples that tested positive for ctDNA ≤ 6 months prior to disease progression were included. N is depicted above each strip, corresponding mean ctDNA concentration is indicated using arrow heads.

Supplementary Material 4 Sequences for primers and probes. Fluorescein (FAM)- or hexafluorescein (HEX)-labeled probes with locked nucleic acid (LNA) were designed using Beacon Designer Version 8.20 (Premier Biosoft, Palo Alto CA, USA). Primers and probes were designed by IDT DNA Technologies, Inc. (Coraville IA, USA).

Supplementary Material 5 Sequences for gBlock

Supplementary Material 6 LOB and LOD values. The limit of blank (LOB) values for each assay was determined using cfDNA obtained from healthy individuals and dilutions of DNA containing known mutations. The limit ofblank value was estimated by measuring replicates of a blank sample and calculating the mean result and the standard deviation (SD): LoB = meanblank + 1.645(SDblank). Thus, the LOB denotes false positivity or specificity of the assay. LoD was determined by utilising both the measured LoB and test replicates of a sample known to contain a low concentration of analyte: LoD = LoB + 1.645(SD low concentration sample).Thus, LoD is the lowest analyte concentration likely to be reliably distinguished from the LoB and at which detection is feasible and approximates to the analytical sensitivity of the assay. (*p ≤ 0.05; **p ≤ 0.01)

## Data Availability

The dataset generated during and/or analysed during the current study are available from the corresponding author on reasonable request.

## Declarations

### Prior presentation

An abstract containing part of the submitted data has been presented as poster at the DGHO Meeting in 2022 and at the ADO meeting in 2022.

## References

[CR1] Ansstas G, Khaddour K, Sudhaman S et al (2026) Longitudinal ctDNA monitoring for post-surgical disease surveillance in patients with stage I-IIIB melanoma. Clin Cancer Res. 10.1158/1078-0432.CCR-25-3643 41632449 10.1158/1078-0432.CCR-25-3643 PMC13080317

[CR2] Boerlin A, Bellini E, Turko P, Cheng PF, Levesque MP, Dummer R, Ramelyte E (2022) The prognostic value of a single, randomly timed circulating tumor dna measurement in patients with metastatic melanoma. Cancers (Basel). 10.3390/cancers14174158 36077695 10.3390/cancers14174158 PMC9455041

[CR3] Bratman SV, Yang SYC, Iafolla MAJ et al (2020) Personalized circulating tumor DNA analysis as a predictive biomarker in solid tumor patients treated with pembrolizumab. Nat Cancer 1:873–881. 10.1038/s43018-020-0096-5 35121950 10.1038/s43018-020-0096-5

[CR4] Braune J, Keller L, Schiller F et al (2020) Circulating tumor DNA allows early treatment monitoring in BRAF- and NRAS-mutant malignant melanoma. JCO Precis Oncol 4:20–31. 10.1200/PO.19.00174 35050727 10.1200/PO.19.00174

[CR5] Brunsgaard EK, Bowles TL, Asare EA et al (2023) Feasibility of personalized circulating tumor DNA detection in stage II and III melanoma. Melanoma Res 33:184–191. 10.1097/CMR.0000000000000892 37040662 10.1097/CMR.0000000000000892 PMC10144272

[CR6] Buja A, Rugge M, Trevisiol C et al (2024) Cutaneous melanoma in adolescents and young adults. J Eur Acad Dermatol Venereol 38:1997–2004. 10.1111/jdv.20077 38709156 10.1111/jdv.20077

[CR7] Chan WY, Lee JH, Stewart A et al (2024) Circulating tumour DNA dynamics predict recurrence in stage III melanoma patients receiving neoadjuvant immunotherapy. J Exp Clin Cancer Res 43:238. 10.1186/s13046-024-03153-1 39169411 10.1186/s13046-024-03153-1 PMC11337884

[CR8] Chang GA, Tadepalli JS, Shao Y et al (2016) Sensitivity of plasma BRAFmutant and NRASmutant cell-free DNA assays to detect metastatic melanoma in patients with low RECIST scores and non-RECIST disease progression. Mol Oncol 10:157–165. 10.1016/j.molonc.2015.09.005 26440707 10.1016/j.molonc.2015.09.005 PMC4695284

[CR9] Ertekin SS, Podlipnik S, Ribero S et al (2020) Monthly changes in serum levels of S100B protein as a predictor of metastasis development in high-risk melanoma patients. J Eur Acad Dermatol Venereol 34:1482–1488. 10.1111/jdv.16212 31967695 10.1111/jdv.16212

[CR10] Gandini S, Zanna I, De Angelis SP et al (2021) Circulating tumour DNA and melanoma survival: a systematic literature review and meta-analysis. Crit Rev Oncol Hematol 157:103187. 10.1016/j.critrevonc.2020.103187 33276181 10.1016/j.critrevonc.2020.103187

[CR11] Heidrich I, Deitert B, Werner S, Pantel K (2023) Liquid biopsy for monitoring of tumor dormancy and early detection of disease recurrence in solid tumors. Cancer Metastasis Rev 42:161–182. 10.1007/s10555-022-10075-x 36607507 10.1007/s10555-022-10075-x PMC10014694

[CR12] Keegan THM, Abrahao R, Alvarez EM (2024) Survival trends among adolescents and young adults diagnosed with cancer in the United States: comparisons with children and older adults. J Clin Oncol 42:630–641. 10.1200/JCO.23.01367 37883740 10.1200/JCO.23.01367 PMC12040215

[CR13] Khaddour K, Zhou A, Butt OH, Budde G, Malashevich AK, Ansstas G (2022) Case report: real-world experience using a personalized cancer-specific circulating tumor DNA assay in different metastatic melanoma scenarios. Front Oncol 12:978996. 10.3389/fonc.2022.978996 36465349 10.3389/fonc.2022.978996 PMC9713015

[CR14] Le Guin CHD, Bornfeld N, Bechrakis NE, Jabbarli L, Richly H, Lohmann DR, Zeschnigk M (2021) Early detection of metastatic uveal melanoma by the analysis of tumor-specific mutations in cell-free plasma DNA. Cancer Med 10:5974–5982. 10.1002/cam4.4153 34291585 10.1002/cam4.4153 PMC8419753

[CR15] Lee RJ, Gremel G, Marshall A et al (2018) Circulating tumor DNA predicts survival in patients with resected high-risk stage II/III melanoma. Ann Oncol 29:490–496. 10.1093/annonc/mdx717 29112704 10.1093/annonc/mdx717 PMC5834029

[CR16] Lee JH, Menzies AM, Carlino MS et al (2020) Longitudinal monitoring of ctDNA in patients with melanoma and brain metastases treated with immune checkpoint inhibitors. Clin Cancer Res 26:4064–4071. 10.1158/1078-0432.CCR-19-3926 32321716 10.1158/1078-0432.CCR-19-3926

[CR17] Long GV, Swetter SM, Menzies AM, Gershenwald JE, Scolyer RA (2023) Cutaneous melanoma. Lancet 402:485–502. 10.1016/S0140-6736(23)00821-8 37499671 10.1016/S0140-6736(23)00821-8

[CR18] Marchisio S, Ricci AA, Roccuzzo G et al (2024) Monitoring circulating tumor DNA liquid biopsy in stage III BRAF-mutant melanoma patients undergoing adjuvant treatment. J Transl Med 22:1074. 10.1186/s12967-024-05783-7 39609824 10.1186/s12967-024-05783-7 PMC11603725

[CR19] Marczynski GT, Laus AC, Dos Reis MB, Reis RM, Vazquez VL (2020) Circulating tumor DNA (ctDNA) detection is associated with shorter progression-free survival in advanced melanoma patients. Sci Rep 10:18682. 10.1038/s41598-020-75792-1 33122747 10.1038/s41598-020-75792-1 PMC7596487

[CR20] Polivka J, Gouda MA, Sharif M et al (2024) Predictive significance of combined plasmatic detection of BRAF mutations and S100B tumor marker in early-stage malignant melanoma. Cancer Med 13:e70313. 10.1002/cam4.70313 39387479 10.1002/cam4.70313 PMC11465285

[CR21] R Development Core Team (2021) R: a language and environment for statistical computing. R Foundation for Statistical Computing, Vienna, Austria

[CR22] Schmid-Wendtner MH, Baumert J, Eberle J, Plewig G, Volkenandt M, Sander CA (2003) Disease progression in patients with thin cutaneous melanomas (tumour thickness < or = 0.75 mm): clinical and epidemiological data from the Tumour Center Munich 1977–98. Br J Dermatol 149:788–793. 10.1046/j.1365-2133.2003.05599.x 14616371 10.1046/j.1365-2133.2003.05599.x

[CR23] Stadler JC, Belloum Y, Deitert B et al (2022) Current and future clinical applications of ctDNA in immuno-oncology. Can Res 82:349–358. 10.1158/0008-5472.CAN-21-1718 10.1158/0008-5472.CAN-21-1718 PMC9397642 34815256

[CR24] Syeda MM, Long GV, Garrett J et al (2025) Clinical validation of droplet digital PCR assays in detecting BRAF(V600)-mutant circulating tumour DNA as a prognostic biomarker in patients with resected stage III melanoma receiving adjuvant therapy (COMBI-AD): a biomarker analysis from a double-blind, randomised phase 3 trial. Lancet Oncol 26:641–653. 10.1016/S1470-2045(25)00139-1 40250457 10.1016/S1470-2045(25)00139-1

[CR25] Tan L, Sandhu S, Lee RJ et al (2019) Prediction and monitoring of relapse in stage III melanoma using circulating tumor DNA. Ann Oncol 30:804–814. 10.1093/annonc/mdz048 30838379 10.1093/annonc/mdz048 PMC6551451

[CR26] Wong SQ, Raleigh JM, Callahan J et al (2017) Circulating tumor DNA analysis and functional imaging provide complementary approaches for comprehensive disease monitoring in metastatic melanoma. JCO Precis Oncol 1:1–14. 10.1200/PO.16.00009 35172485 10.1200/PO.16.00009

