# Supplementary material for: Sensitivity and prognostic significance of circulating tumor DNA (ctDNA) in stage I to III malignant melanoma
Source: J Cancer Res Clin Oncol. 2026 May 9;152(5):106. doi: 10.1007/s00432-026-06478-w (PMC13168401; doi:10.1007/s00432-026-06478-w)
Supplement: Supplementary file 1 — Supplementary Material 1 Patients were divided into two subgroups. One subgroup including patients with BRAF or NRAS mutated primary tumors and one subgroup with wildtype BRAF and NRAS primary tumors. Following purification of ctDNA the BRAF+/NRAS+ subgroup samples were examined for presence of BRAF+/NRAS+ctDNA. Following purification of ctDNA the wildtype subgroup samples were examined for presence of TERT promoter mutated ctDNA. [file 432_2026_6478_MOESM1_ESM.pdf]

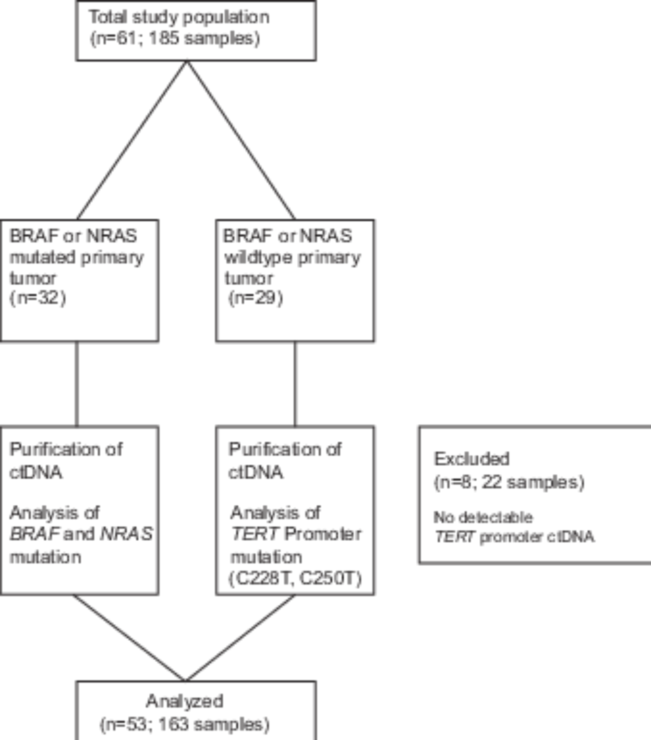

Total study population
(n=61; 185 samples)
BRAF or NRAS mutated primary tumor (n=32)
BRAF or NRAS wildtype primary tumor (n=29)
Purification of ctDNA
Analysis of *BRAF* and *NRAS* mutation
Purification of ctDNA
Analysis of *TERT* Promoter mutation (C228T, C250T)
Excluded
(n=8; 22 samples)
No detectable *TERT* promoter ctDNA
Analyzed
(n=53; 163 samples)
