# Supplementary figures and images for: Sensitivity and prognostic significance of circulating tumor DNA (ctDNA) in stage I to III malignant melanoma
Source: J Cancer Res Clin Oncol. 2026 May 9;152(5):106. doi: 10.1007/s00432-026-06478-w (PMC13168401; doi:10.1007/s00432-026-06478-w)

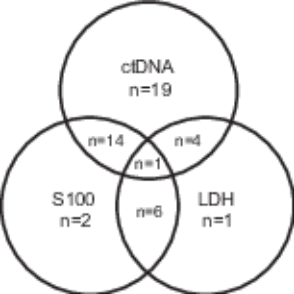

Supplement: Supplementary file 2 — Supplementary Material 2 Each biomarker (ctDNA, S100, LDH) is indicated by a circle. Overlapping areas of the circles of the diagram represent overlap in biomarker positivity in the indicated number of patients. [file 432_2026_6478_MOESM2_ESM.pdf]

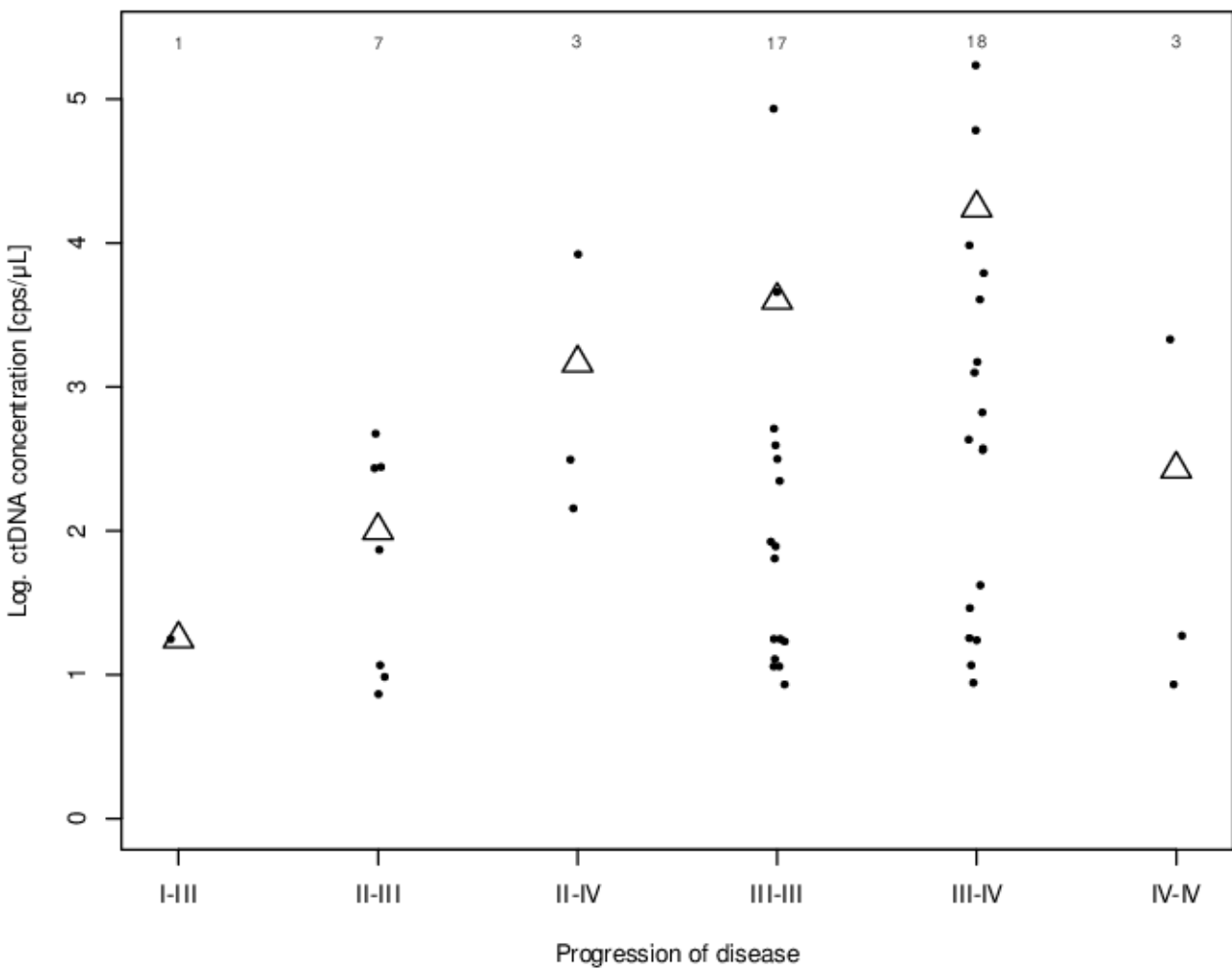

Supplement: Supplementary file 3 — Supplementary Material 3 Strip chart depicting the logarithmic (LOG) concentration of ctDNA (y-axis) dependent on shifts between AJCC stages (x-axis). Only samples that tested positive for ctDNA ≤ 6 months prior to disease progression were included. N is depicted above each strip, corresponding mean ctDNA concentration is indicated using arrow heads. [file 432_2026_6478_MOESM3_ESM.pdf]
