# Supplementary material for: Sensitivity and prognostic significance of circulating tumor DNA (ctDNA) in stage I to III malignant melanoma
Source: J Cancer Res Clin Oncol. 2026 May 9;152(5):106. doi: 10.1007/s00432-026-06478-w (PMC13168401; doi:10.1007/s00432-026-06478-w)
Supplement: Supplementary file 4 — Supplementary Material 4 Sequences for primers and probes. Fluorescein (FAM)- or hexafluorescein (HEX)-labeled probes with locked nucleic acid (LNA) were designed using Beacon Designer Version 8.20 (Premier Biosoft, Palo Alto CA, USA). Primers and probes were designed by IDT DNA Technologies, Inc. (Coraville IA, USA). [file 432_2026_6478_MOESM4_ESM.pdf]

Table ESM_4

	LNA WT	LNA MUT	Sense Primer	Antisense Primer
BRAF V600E	5‘-tcgaGatTtcAc tGtagct-3‘	5‘- tcgaGatTtcTct Gtagct-3‘	5‘- GATCCAGACAACTGT TCA-3‘	5‘- ACACCTCAGATATAT TTCTTCA-3‘
NRAS Q61K	5‘- tacTctTgtTgt Ccagct-3‘	5‘- tacTctTctTatCc agctg-3‘	5‘- GCCTGTCCTCATGTA TTG-3‘	5‘- AGTGGTTATAGATG GTGAAAC-3‘
NRAS Q61R	5‘- tacTctTgtTgt Ccagct-3‘	5‘- ctctTctCgtCca Gctg-3‘	5‘- GCCTGTCCTCATGTA TTG-3‘	5‘- AGTGGTTATAGATG GTGAAAC-3‘
NRAS Q61L	5‘- tacTctTgtTgt Ccagct-3‘	5‘- tacTctTctAgtCc agct-3	5‘- GCCTGTCCTCATGTA TTG-3‘	5‘- AGTGGTTATAGATG GTGAAAC-3‘
BRAF V600K	5‘- ctaGctAcaGt gAaatctc-3‘	5‘- tagCtaCaaAga Aatctcg-3‘	5‘- CACCTCAGATATATT TCTTCATG-3‘	5‘- GATCCAGACAACTG TTCAA-3‘
