# Supplementary material for: Sensitivity and prognostic significance of circulating tumor DNA (ctDNA) in stage I to III malignant melanoma
Source: J Cancer Res Clin Oncol. 2026 May 9;152(5):106. doi: 10.1007/s00432-026-06478-w (PMC13168401; doi:10.1007/s00432-026-06478-w)
Supplement: Supplementary file 5 — Supplementary Material 5 Sequences for gBlock [file 432_2026_6478_MOESM5_ESM.pdf]

Table ESM_5

Mutation	gBlock
BRAF V600K	5‘-<br>GTTTTAAAGAATATTATATTACAGAATTATAGAAATTAGATCTCTTACCTAAACTCTTCATAATGCTTGCTCTGATAGGAAAATGAGATC<br>TACTGTTTTCCTTTA<br>AAATCTCGATGGAGTGGGTCCCATCAGTT<br>ATTAAATTTTTGGCCCTGAGATGCTGCTGAGTTACTAGAAAGTCA<br>CAAAA-3‘
NRAS Q61K	5‘-<br>ACAACCTAAAACCAACTCTTCCCATAATTAAAAAGCTCTATCTTCCCTAGTGTGGTAACCTCATTTCCCCATAAAGATTCAGAACACA<br>AAGATCATCCTTTCAG<br>CAGAGGAAGCCTTCGCCTGTCCTCATGTAT<br>AGGTTTCACCATCTATAACCACTTGTTTTCTGTAAGAATCCTGGG<br>TTTTATTAAAAACCACAGGGAATGCAATGCTATTGCCAAGGTTAAATAAGCATCTAACTA<br>GTCCCTCAAATTGCTAATATATAATCACAAACAAAAAGTATCCAATATCACCCTAC-3‘
NRAS Q61R	5‘-<br>ACAACCTAAAACCAACTCTTCCCATAATTAAAAAGCTCTATCTTCCCTAGTGTGGTAACCTCATTTCCCCATAAAGATTCAGAACACA<br>AAGATCATCCTTTCAG<br>CAGAGGAAGCCTTCGCCTGTCCTCATGTAT<br>AGGTTTCACCATCTATAACCACTTGTTTTCTGTAAGAATCCTGGG<br>TTTTATTAAAAACCACAGGGAATGCAATGCTATTGCCAAGGTTAAATAAGCATCTAACTA<br>GTCCCTCAAATTGCTAATATATAATCACAAACAAAAAGTATCCAATATCACCCTAC-3‘
NRAS Q61L	5‘-<br>ACAACCTAAAACCAACTCTTCCCATAATTAAAAAGCTCTATCTTCCCTAGTGTGGTAACCTCATTTCCCCATAAAGATTCAGAACACA<br>AAGATCATCCTTTCAG<br>CAGAGGAAGCCTTCGCCTGTCCTCATGTAT<br>AGGTTTCACCATCTATAACCACTTGTTTTCTGTAAGAATCCTGGG<br>TTTTATTAAAAACCACAGGGAATGCAATGCTATTGCCAAGGTTAAATAAGCATCTAACTA<br>GTCCCTCAAATTGCTAATATATAATCACAAACAAAAAGTATCCAATATCACCCTAC-3‘
