# Supplementary material for: Sensitivity and prognostic significance of circulating tumor DNA (ctDNA) in stage I to III malignant melanoma
Source: J Cancer Res Clin Oncol. 2026 May 9;152(5):106. doi: 10.1007/s00432-026-06478-w (PMC13168401; doi:10.1007/s00432-026-06478-w)
Supplement: Supplementary file 6 — Supplementary Material 6 LOB and LOD values. The limit of blank (LOB) values for each assay was determined using cfDNA obtained from healthy individuals and dilutions of DNA containing known mutations. The limit ofblank value was estimated by measuring replicates of a blank sample and calculating the mean result and the standard deviation (SD): LoB = meanblank + 1.645(SDblank). Thus, the LOB denotes false positivity or specificity of the assay. LoD was determined by utilising both the measured LoB and test replicates of a sample known to contain a low concentration of analyte: LoD = LoB + 1.645(SD low concentration sample).Thus, LoD is the lowest analyte concentration likely to be reliably distinguished from the LoB and at which detection is feasible and approximates to the analytical sensitivity of the assay. (*p ≤ 0.05; **p ≤ 0.01) [file 432_2026_6478_MOESM6_ESM.pdf]

Table ESM_6

Mutation-Assay	Limit of Blank (LOB) (copies per μL)	Limit of Detection (copies per μL)
BRAF V600E	0.0	1:4833**
BRAF V600K	2.2	1:5348**
NRAS Multiplex	0	Q61K: 1:2513*<br>Q61L: 1:10178*<br>Q61R: 1:4573*
TERT C228T	5.1	1:1160*
TERT C250T	2.7	1:3946*
